# Peer Review of “Real-Time Health Monitoring Using 5G Networks: Deep Learning–Based Architecture for Remote Patient Care”

**DOI:** 10.2196/83424

**Published:** 2025-10-01

**Authors:** Francisco Javier Gonzalez-Canete

**Affiliations:** 1 Universidad de Malaga, Avda. Cervantes, Malaga, Spain, 34 952 13 10 00

**Keywords:** 5G, real-time patient monitoring, vital signs, prediction, deep learning, machine learning

*This is a peer-review report for “Real-Time Health Monitoring Using 5G Networks: Deep Learning–Based Architecture for Remote Patient Care.”*

## Round 1 Review

### General Comments

This paper [1] proposes an architecture that integrates a deep learning method with 5G networks to monitor health parameters. These parameters are analyzed using a deep learning system located in the edge network. The decisions taken from the edge system are transfered to a medical server using the 5G network. The objective is to obtain a high-accuracy evaluation of the deep learning system and obtain very low latency in data transmission and reception.

### Specific Comments

#### Major Comments

1. Table 3 is not referenced nor commented on in the text. You should add a paragraph explaining the table or delete it.
2. Table 5 compares the system performance with 3 other systems, A, B, and C, but those systems are never described. They must be commented on in order to compare results.

#### Minor Comments

1. Equation 1 has no label (1) and it is defined twice.
2. Figure 4 should be placed after it is called out.
3. On page 6, there is a sentence in square brackets.
4. Correct the sentence “Figure 4illustrates...” The number 4 and the word “illustrates” are too close.
5. Table 5 is called out before Table 4. Consequently, they should be switched.
6. The sentence “Table V System Comparison...” seems to be a figure description instead of part of the text. It makes no sense in the place it is located.
7. The text “(*P* ! .001)” I presume should be “(*P*<.001)”

## Round 2 Review

### General Comments

This paper presents an architecture to perform real-time monitoring of health signals using 5G networks and deep-learning prediction of possible health problems using the aquired signals.

### Specific Comments

#### Major Comments

1. There are some equations with no defined parameters. In equation 16, what are *Pij* and *xij*? In equation 17, what is *N*? In equation 18, what are *Bi*, *Cj*, and *M*? In equation 19, what is *Lu*? They must be defined.
2. How are weights *wu*, *wr*, and *wl* calculated or estimated? What are their chosen values? The final performance could change depending on the selection of these parameters, as you are giving more importance to one parameter or another.

#### Minor Comments

1. Most of the references are “touching” the previous text. Add a space between text and references. For instance: “...clinical settings[1],[2].” should be “... clinical settings [1], [2].”
2. Figure 2 should be closer to where it is referred to on the previous page.
